# The Experiences of People with Diabetes during COVID-19 Pandemic Lockdown

**DOI:** 10.3390/ijerph19010340

**Published:** 2021-12-29

**Authors:** Modi Al-Moteri, Virginia Plummer, Hanan A. M. Youssef, Ruba W. H. Yaseen, Mohammed Al Malki, Ahmed AbdElbagy Ibrahim Elryah, Ahmed Al Karani

**Affiliations:** 1 Nursing Department, College of Applied Medical Sciences, Taif University, P.O. Box 11099, Taif 21944, Saudi Arabia; dr_h_911@hotmail.com (H.A.M.Y.); Ruba-n-h@hotmail.com (R.W.H.Y.); mhdalmalki51@gmail.com (M.A.M.); ibrahem281@gmail.com (A.A.I.E.); asakg@live.com (A.A.K.); 2 School of Health, Federation University, Mount Helen, VIC 3350, Australia; v.plummer@federation.edu.au

**Keywords:** diabetes mellitus, lockdown, sugar control, vaccine

## Abstract

Little is known about the theoretical foundation underling the response of people with diabetes managing their everyday routines during COVID-19 pandemic lockdown. Aim: To explore the experience of people with diabetes during COVID-19 pandemic lockdown in light of the risk perception, response and behavioral change theories. Method: A qualitative descriptive design was employed, and Braun and Clark’s six step analysis were used for thematic analysis. Semi-structured interviews were conducted online using Zoom Videos Communication. Result: Five themes were defined as follows: (1) perceived the threat and faced their fears, (2) appraised the damage, (3) identified the challenges, (4) modified their routine, and (5) identified the strengths that facilitate the efficacy of their response. There were eight sub-themes within the themes. Conclusion: The results of this study may provide an opportunity for nurses to reflect on issues highlighted by the patients regarding more effective communication, knowledge and skill development for people to support self-care during national emergencies.

## 1. Introduction

In December 2019, in Wuhan city, China, the first case of COVID-19 was reported [1]. The World Health Organization (WHO) soon declared the international spread of COVID-19 a global crisis. There was a lack of preparedness in managing the public health emergency in the first half of 2020 [2] and, in response to the rising number of coronavirus cases, many countries worldwide implemented total or intermittent lockdown to restrict movement and suppress infection. While social distancing was a strategy that has saved many lives, lockdowns enforced many restrictions on the public.

In the Kingdom of Saudi Arabia, lockdown was imposed on 24 March 2020 and eased in July 2020 [3]. During the 91-day lockdown, the quality of life for people with diabetes was negatively impacted [4]. There was a loss of continuum of care, along with an unsuitable impact on lifestyle which caused physical and psychological stress for people with diabetes [5]. Indeed, living under lockdown, people with diabetes may experience barriers to self-management. Alqahtani et al. [6], Alshareef et al. [4] and Banerjee et al. [7] demonstrated that inaccessibility to medical services, reduced physical activity and a sedentary lifestyle, change in eating habits, anxiety and sleep disturbance have all posed challenges to adequate diabetes management. 

## 2. Theoretical Background

Lockdown is the same, however, people have responded differently around the globe [8]. Whilst some people with diabetes find it easy to adapt coping strategies to control their health during the lockdown with great care, others find it too difficult. These differences may be accounted for a number of variables, such as social, cultural and economic factors. Several theories have been developed to help researchers understand how people accept and adapt lifestyle changes to avoid risk, including the protection-motivation theory (PMT), [9] health belief model, [10] and the fear-drive theory [11]. These behavioral change theories build upon basic assumption of “risk perception and response”.

Risk perception is one of the most powerful factors that can cause change in people’s lifestyle and health behaviors [12]. Rogers’ protection-motivation theory (PMT) has provided a conceptual explanation to understand the cognitive processes underlying behavior change [12]. According to PMT, people will select to adapt or to maladapt coping strategies after encountering risk. The perception of the danger will motive individuals to protect themselves through assessing the available coping strategies. The more significant the “protection motivation”, the more behavioral change. 

The health belief model [9] describes two main components of health behavior: threat (including perception of vulnerability to risk) and outcome expectations. In the case of the risks associated with lockdown due to the COVID-19 pandemic, threat could be the susceptibility to a fluctuating blood glucose. The outcome is the perceived benefits of modified self-management, such as measuring blood glucose more frequently as a measure to prevent developing complications due to lockdown.

According to the third model—fear-drive theory—Janis, Ref. [13] stated that fear motivates individuals to adopt change to relieve the negative consequences of the risk. When fear is produced in response to a risk, the “need to reduce fear” feeling causes a change in behavior. The model claimed that, the greater the fear, the greater the possibility that the fear reduction strategies will be successfully adapted; however, excessive fear will cause a decrease in behavioral changes. 

Based on the theoretical perspectives of these three behavioral change theories, there are four basic principles which serve the basic theoretical assumption of “risk perception and response”: two related to evaluations of risk, and two related to evaluation of the proposed responses. In lockdown, people with diabetes may perceive a risk that can alter their ability to manage and control their diabetes on a daily basis. They may start to search for alternative solutions (modified self-management routine) and evaluate the effectiveness of such solutions to protect themselves from the danger of the consequences of an inability to control their blood glucose. This may come under four conditions:

1. Perceived risk: how serious will the risk be if the person does not apply preventive measures, e.g., lockdown and vaccine, for their health?
2. Perceived probability of damage: how possible is it that their regular self-management routine might be affected by the lockdown?
3. Perceived response efficacy: how effective do they think the alternative solutions (e.g., modified self-management routine) will be in their lives?
4. Perceived self-efficacy: how confident do they feel that they able to adapt alternative solutions (e.g., modified self-management routine) under the lockdown?

To summarize, it can be said that threats or risks arising from lockdown may make people face their fears, believing that they can handle the threat and focus on alternative solutions when responding.

Studies conducted to investigate how people with diabetes lived during the experience of COVID-19 pandemic lockdown are scant [14,15] and lack a theoretical foundation. Of these studies, a recent study conducted by Pal et al. [16] found that most of the participants being interviewed experienced a reduction in physical activity associated with worsening of blood glucose control during the lockdown. The researchers recommended increasing diabetes self-management awareness through conducting planned educational programs [16]. In another recent qualitative study, participants handled their daily routine changes during the lockdown experiences in very different ways [17]. Similar to Pal et al. [16], Grabowski et al. [17] confirmed the need for guidance and support during lockdown. This implies the importance of conducting more studies to contribute to the body of knowledge and significant understanding of how people with diabetes manage their life in emergencies, disasters or pandemics. Indeed, this will help in developing plans and guidelines to support people with diabetes.

The basic theoretical assumption of “risk perception and response” for changing behavior during the lockdown is very important to understanding how people faced the challenges in maintaining their life under a pandemic [11]. However, limited studies have investigated the impact of this theoretical assumption on promoting behavioral changes during the COVID-19 lockdown for patients with diabetes. Recognizing the existence of a gap in knowledge, this study aims to explore the experience of people with diabetes during COVID-19 pandemic lockdown in light of risk perception and response, and behavioral change theories.

## 3. Methods

An explorative descriptive qualitative design was selected using semi-structured interviews to better understand how people with diabetes managed their life during the COVID-19 lockdown

### 3.1. Setting and Participants

The study invited adult patients with type 1 or 2 diabetes mellitus with a mobile number recorded in the electronic medical records at a Diabetes Center in the western region of Saudi Arabia. It was found that 1234 patients with diabetes were registered in the Diabetes Center, of all ages and of both Saudi and non-Saudi background. The Diabetes Center is equipped with sufficient medical staff taking care of people with diabetes, including 11 consultants and diabetic specialists, and 20 nurses (males and females). Medical staff are highly trained. The Diabetes Center provides all types of medical, follow up and educational services for people with diabetes, regardless of age gender, background and type of diabetes including type 1, type 2, impaired glucose tolerance and gestational diabetes mellitus.

In addition to a diagnosis of type 1 or 2 diabetes, the inclusion criteria involved adult patients who are able to use social media. The exclusion criteria were children, women with gestational diabetes mellitus, being diagnosed during or shortly before the lockdown (less than six months) and suffering from critical comorbidities, such as renal and cardio-cerebrovascular complications. 

An automated text message was sent out via mobile telephones inviting those people with diabetes who were registered in the Diabetes Center to participate in a one-on-one interview. The decision to use virtual one-on-one interviews instead of telephone interviews came after a long discussion between the authors. The fact that the virtual one-on-one interviews could replicate the experience of face-to-face interviews [18], prompted the researchers to select this approach for data collection initially. Two key features made the Zoom platform preferable for data collection compared to other web conferencing platforms: the simplicity and the ease of use. Participant can log into Zoom without the need to download any application or a create Zoom account. Participants only needed to join the meeting through a previously sent private meeting link.

**Figure A1:**
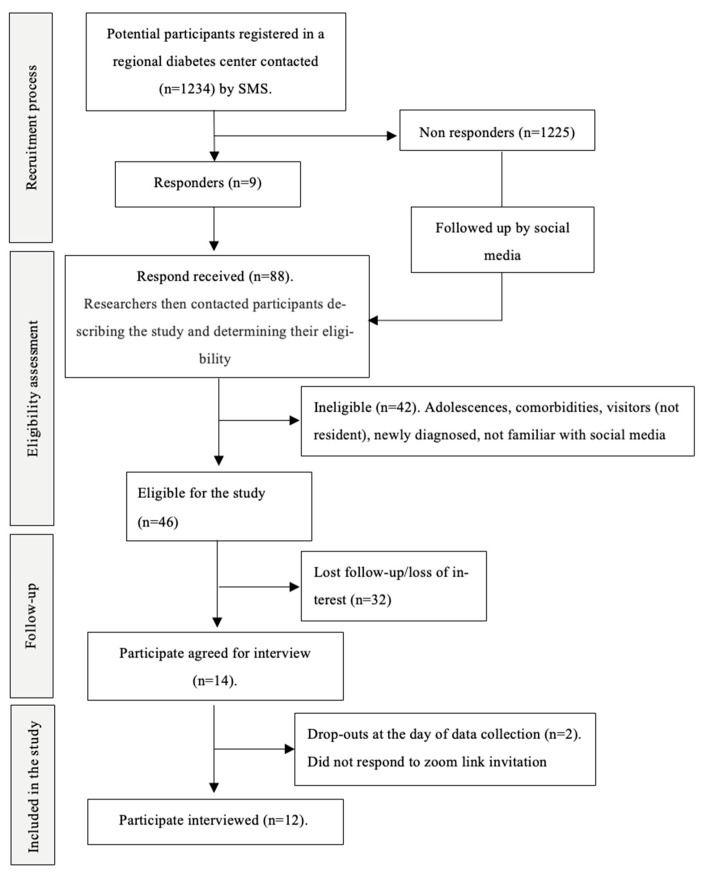
Recruitment Flowchart.

Initially recruitment was slow, resulting in an insufficient number of responders. As a result, a follow up on non-responders began through considering social media platforms (Twitter and WhatsApp). The purpose of qualitative studies is to explore a particular aspect of behavior relevant to a group of people who live in conditions pertinent to the phenomenon being studied, rather than to determine a representative sample drawn from a population [19]. This allows researchers to select a specific recruitment approach and inclusion criteria (Recruitment flowchart, Appendix A Figure A1). 

Interested participants were then contacted to explain the study, confirm eligibility and set a meeting date and time. 

### 3.2. Interviews

According to Guest, Bunce and Johnson [20], 10–15 interviews are adequate to achieve saturation and identify all of the main themes. Data collection took place over three weeks in July 2020. A qualitative research guide was developed by the researchers based on the literature [21] and was used to facilitate the interview. The guide explored the health management of people with diabetes during the lockdown. 

Due to the COVID-19 lockdown, MM and HA conducted all of the virtual one-on-one interviews using Zoom Videos Communication. Twelve individual semi-structured online interviews were conducted. 

Prior to conducting the interviews, information was provided to the participants about the purpose of the study and the ethical approval. Verbal consents were obtained, and participants were informed that participation was voluntary. Participants were probed, as necessary, to help them to expand on their answers. 

The interviews took from 20 to 30 min and were digitally recorded and started by asking an open question about the interviewees’ thoughts concerning managing their life during the COVID-19 lockdown. Specific attention was given to three general topics by the two interviewers MM and HA: information about routine diabetes care and changes during the COVID-19 lockdown; diabetes-related worry and concern; and COVID-19 vaccine perception (Interviews protocol, Appendix B).

Probing questions, such as “tell us how you maintained a healthy lifestyle during the COVID-19 lockdown?”; “did your activity change during the COVID-19 lockdown? If so, how?”; “were you worried about your health during the COVID-19 pandemic? Does this affect your life?”; and “what do you think about COVID-19 vaccine?” were used. At the end of the interview, researchers asked interviewees if there was anything more that they would like to add, or if they had any questions they would like to ask. Researchers then thanked interviewees for their participation.

## 4. Analysis

Braun and Clark’s six step analysis were used for thematic analysis [22]. The six steps approach is an explorative descriptive approach for qualitative data. In the first step, the authors (MM and HA) familiarized themselves with the data by reading and rereading the interview transcripts line by line to search for meanings and patterns, and to obtain a general understanding. As the researchers read through the transcripts, they coded words and phrases or elements that can have a meaning regarding diabetes self-management during lockdown. Meanwhile, researchers’ thoughts were individually recorded in a memo for later use. In the second step, initial codes were segmented. In the third step the relevant segments were sorted and collated into potential themes. In the fourth step, researchers reviewed and refined themes through reading the collated extracts for each theme to form a coherent pattern Researchers met frequently to compare and discuss their findings and the interpretations of the identified codes. In step five, the researchers started to define and name the themes to identify the essence of what each theme captured. To check the representativeness and the accuracy of the identified themes, one researcher (HA) cross-checked with the key themes and sub-themes and the interviewees verbatim. This step is essential to increase the rigor of the findings. Findings were then organized and presented based on the risk perception and response and behavioral change theories. 

Demographic data were described using means and standard deviations (e.g., age, duration of diabetes). 

### 4.1. Data Rigour

Data rigor was maintained through establishing the credibility, transferability, dependability and conformability of the data [23]. Peer checking was undertaken to ensure credibility through examining the transcripts by the entire research team. Any doubts were then resolved. Some participants were invited to judge the end results of the analysis. Efforts were made to present the findings through the participants’ eyes and to maintain the cultural context and societal setting of Saudi Arabia to ensure credibility of the data. This was essential to maintain transferability. All study steps were carefully planned to guarantee dependability. An interview protocol was established to maintain consistency during the interview. Finally, conformability was maintained, through careful analysis, to ensure that the themes accurately represented the subjective data. 

### 4.2. Findings

#### 4.2.1. Participants

Three women and nine men consented and completed the interview, of which four had type I diabetes and eight had type 2 diabetes (Table 1). Their ages ranged from 22 to 61 years old, with the mean age being 42 years old and the standard deviation being 12.35. The length of their diabetes ranged from 2 to 26 years, with the mean being 10.66 years and the standard deviation being 7.52.

#### 4.2.2. Themes and Subthemes

Five themes and eight subthemes were identified from the thematic analysis and these are presented in Table 2. The themes describe the experiences of people with diabetes during the COVID-19 pandemic lockdown and their efforts to maintain a healthy lifestyle. Because this is an explorative descriptive qualitative study, the qualitative and demographic results were displayed side-by-side in Table 1, in order to identify the specific differences between people with type 1 and type 2 diabetes, with respect to the subthemes. This revealed that developing high blood glucose and complications were a major concern for people with type 1 diabetes, and maintaining physical activity was the main concern for people with type 2 diabetes. 

The following section discusses each theme and its related subthemes along with the relevant example from participants’ interviews.

### 4.3. Theme 1-Facing Their Fears

People with diabetes are more at risk of developing severe symptoms, serious health problems or dying from COVID-19 infection than those without diabetes. Even though, in general, the interviewees expressed few worries about their health under the lockdown, several areas of fear were mentioned. There was fear of infection with COVID-19 and a fear of mandatory vaccination.

*“I was worried…you know listening to news about COVID-19 irritates me a lot… they said our [diabetic people] immune system is weak … this creates a worry in me… I thought okay I will get infection as soon as I go out… I should not leave home…”*

(P-10-type-2).

*“…it is terrible…I heard that you could lose your life if you become infected (with COVID-19)…”*

(P-12-type-1).

COVID-19 vaccine was seen as a major threat, associated with fear. The interviewees were afraid of mandatory vaccination and the possible consequences. Some of the interviewees said that if they had to take it, they would accept it:

*“…honestly I’m scared a lot… I heard that it [COVID-19 vaccine] might be mandatory for people like us [people with diabetes] …because we are at risk…”*

(P-6-type-1).

*“…Well…I think it (the vaccine) is a choice…I will choose to take it if I will be protecting myself and others”*

(P-1-type-2).

### 4.4. Theme 2–The Possibility of Damage

Some interviewees expressed worries about having diabetes-related complications. They were especially worried about not being able to control blood glucose during the lockdown and the consequences of this.

*“I was a bit worried about it [lockdown]…you know for some of us (diabetes people) it’s going to be horrible if we couldn’t control our blood sugar…diabetes brings other problems”*

(P-4-type-2).

*“What really* *terrified me is having sugar ups and downs* *, but then you* *won’t know how much [insulin] you need”*

(P-5-type-1).

*“…you see we are in a situation [the lockdown] we hardly know when it will end up… very low or high blood sugar can cause problems that sometimes need to be treated in hospital… but again, it seems scary to go to hospital on these days …”*

(P-3-type-1).

*“You know you could lose your vision if you don’t control your blood sugar”*

(P-12-type-1).

### 4.5. Theme 3-The Challenges of Everyday Routines

Several people verbalized that living with diabetes is a challenge that they are faced with every day. Diabetes can be managed by following some simple instructions, such as: (1) regular monitoring of blood glucose, (2) taking prescribed medications, (3) eating healthy food, and (4) regular exercise. However, lockdown brought great challenges to maintaining everyday routine practices. Of those challenges, participants reported the challenge of maintaining balance blood glucose:

*“…it (lockdown) has impacted my life…as I am just sitting for a long time…it has become my habit now. I think balancing sugar-to-physical activity…is a bit more difficult now”*

(P-11-type-2).

*“Getting balance between food and insulin was challenging for me during lockdown”*

(P-9-type-1).

### 4.6. Theme 4-Identifying Strengths

“The family” and “the medical team” for diabetes care are mainly the sources of support which take place within the home. Hence, it is not a disease that individuals can manage alone. Family and social support are key players in diabetes management. During the COVID-19 pandemic and, as reported by some participants, family contribute to easing the stress of the lockdown and ensuring diabetic care:

*“…I was a bit worried at the beginning (of the lockdown)… but I was able to maintain my diabetic routine because of my family…you see, my family has always supported me…”*

(P-12-type-1).

*“…My family has taken care of me very well [during the lockdown]…”*

(P-4-type-1).

Participants also commented on the support they received from their healthcare team during the COVID-19 lockdown. According to the participants, their healthcare teams supported and followed participants, even on weekends:

*“…healthcare team was very helpful and supportive during the lockdown…they [healthcare team] never stop following my health, they are ready when I need them even during weekends.”*

(P-5-type-1).

A considerable number of the interviewees found that the lockdown had given them the time to think about their general wellbeing. This presented an opportunity to change. For instance, the following interviewees commented on how the lockdown had allowed them to think deliberately about their health:

*“…even though it [lockdown] was hard somehow at the beginning, but it gave me plenty of free time to seriously think about my health …”*

(P-8-type-1).

*“….though before lockdown I always went to the medical center for follow up, but never gave time to my thoughts, about my health. During the lockdown I have got time to focus on my health…”*

(P-2-type-2).

For some it was important to search for the necessary information about how to deal with the diabetes during the lockdown. Participants obtained information from websites and the media, which were seen as very valuable information resources.

*“…I started to think about my health…there is lots and lots of trusted channels and websites to get information by yourself telling you how to stay healthy…in such critical time [COVID-19]…you can learn how to eat healthy food…”*

(P-12-type-1).

### 4.7. Theme 5-Finding and Adapting of Alternative Solutions for Everyday Routine

Concerning dietary habits following the lockdown, for some participants it was difficult and a huge challenge, whereas others did not mention any great challenges or problems. Instead, they started to think of the lockdown an opportunity to change their bad dietary habits and focus on healthy food.

*“…* *You know I like junk food very much…this [lockdown]* *has made only one thing right, that is, you know, closing down the fast food restaurants…”*

(P-7-type-2).

*“…I worked hard on changing my diet during the lockdown, … I started to increase the healthy food such as salads, brown bread in my meals…I was afraid of getting high blood sugar…”*

(P-3-type-1).

Changing physical activity was also challenging. The comments made by some participants reported how they engaged in the process of finding an alternative method to maintain their routine physical activities to cope with the lockdown:

*“…well due to the lockdown, my routine has been disturbed. You see I have a tight schedule, but I’ve never missed outside walking…now I am getting time but then also I am unable to go outside for long walk… I missed that…I have made a shift from outside to short yard walking…and found myself accepting that…”*

(P-9-type-2).

*“You know I’m type 2 diabetes…to maintain my weight I was too much dependent on equipment to exercise in the gym. With lockdown there is no option left…but I searched for an alternatives on the internet, I found them using some innovative way, they are using water bottles…I am using that now and these are really helpful…”*

(P-2-type-2).

*“See, as you might know not everyone has exercise equipment at home. So, I increased my physical activity level by doing workouts at home, say stair climbing…”*

(P-8-type-2).

*“well, during the lockdown my activity level slumped, I challenged myself to walk* around *my* house *for one hour every day…”*

(P-5-type-1).

### 4.8. Framework

**Figure 1:**
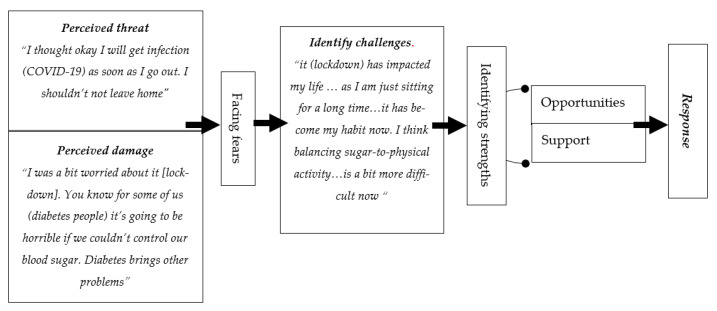
Summary of the study findings in relation to the theoretical assumption.

A framework that was created as a summary of the study findings in relation to the theoretical assumption “risk perception and response” for changing behaviors, to understand the response of people with diabetes during the lockdown. The suggested framework’s structure is displayed in Figure 1 and encompasses many components. The first part of the framework includes the components that contribute to behavior changes (response): perception of the risk and damage possibility. The second part includes facing fears resulting from risk perception. The third part includes the point when people with diabetes start to identify challenges and strength factors. The last part is the response. This was organized as follows: (1) the patient perceived the threat, and faced their fears, (2) the patient appraised the damage, (3) the patient identified the challenges, (4) the patient identified the strengths that facilitated the efficacy of their response, and (5) the patient modified their routine.

## 5. Discussion

While a lot of qualitative studies conducted with people who have diabetes seek to understand their experience, published qualitative studies on how those people managed their life during COVID-19 lockdown within the basic theoretical assumptions of “risk perception and response” and the coping strategies to manage everyday routines are few. These study findings enhance our understanding of how people with diabetes managed issues and concerns resulting from the lockdown in the Kingdom of Saudi Arabia.

In the current study, the results, in general, were consistent with the structure of the theoretical assumption of “risk perception and response”—perception of risk, perception of susceptibility of damage and response (behavioral change). This shows that interviewees recognized COVID-19 and the related lockdown as a significant threat (risk) that could potentially affect their health. Part of this perception, as suggested by, ref. [24] is due to panic emotion, which includes experiences derived from public media and national massages. People with diabetes who were included in the study expressed their fears about the possibility of obtaining a COVID-19 infection, as shown in the comment below:

*“…it is terrible …I heard that you could lose your life if you become infected (with COVID-19)* *…”*

(P-12-type-1).

Similar findings were reported in the literature, as Grabowski et al. (2021) found that interviewees were worried about the risk of become infected with COVID-19. A high prevalence of fears and worries were found among people with diabetes and this requires urgent interventions [25]. News media and social media platforms have played significant roles in increasing the fears and worries among people with chronic disease, especially those with diabetes [26,27], through amplifying their perceptions of risk. This area of investigation should be highlighted in future research

Perceiving the susceptibility of damage resulting from lockdown can directly affect behavioral change and is also consistent with previous studies [28]. Interviewees in this study verbalized their worries as a consequence of the COVID-19 pandemic lockdown on their blood sugar, as shown in the comment below:

*“I was a bit worried about it [lockdown]…you know for some of us (diabetes people) it’s going to be horrible if we couldn’t control our blood sugar…diabetes brings other problems”*

(P-4-type-2).

The relation between worries about possible damage and maintaining blood glucose have been investigated profoundly [29,30,31]. In most of these studies, a negative impact was found on the ability of people with diabetes to manage their illness. Possible damage perception may encourage behavioral change. This can be explained by considering that when people with diabetes believe that they are at risk to develop complications, though they may not adapt preventive measures, they will start to form mental readiness to participate in coping behaviors [28].

The intention to change behaviors in response to lockdown may include identifying challenges, as shown in the comment below:

*“Getting balance between food and insulin was challenging for me during lockdown”*

(P-9-type-1).

Nevertheless, it was note-worthy that some participants reported managing these difficulties by looking at it as an opportunity to change and learn. People with diabetes in the current study started to increase their knowledge and awareness about the preventive and the alternative measures to stay healthy during the lockdown. They adopted and implemented new ways to manage the consequence of the restrictions. Tiwari et al. [32] found that around half of the participants included in their study were exercising and checking their blood glucose regularly, felt good and involved themselves in new hobbies. Furthermore, the study by Tiwari et al. [32] showed that family support serves as a buffer to counteract the negative effects of stress on blood glucose. This was aligned with the findings of the current study as participants stressed how their families contributed to easing the stress of the lockdown. The support of the members of the family is an important factor in maintaining diabetic care. 

Interviewees in the current study expressed how they participated in coping behaviors. The COVID-19 lockdown created challenges for individuals with diabetes, forcing them to stay at home, which restricted their physical activities, resulting in a sedentary lifestyle. Further, healthy food supplies were not always available. A lack of physical activities and healthy food supplies made participants search for alternative solutions

*“Well, during the lockdown my activity level slumped, I challenged myself to walk* around *my* house *for one hour every day…”*

(P-5-type-1).

*“I have made a shift from outside to short backyard walking…and found myself accepting that…”*

(P-9-type-2).

*“I started to increase the healthy food such as salads, brown bread in my meals…”*

(P-3-type-1).

In this area of investigation, Ruiz-Roso, et al. [33] clearly showed the influence of the COVID-19 lockdown on the daily habits of people with diabetes, especially in terms of food and activities and the potential health consequences.

## 6. Implications

The study findings could be used to devise interventions and instructions to support people with diabetes to successfully manage their illness during such crises or any public emergencies. No guidance or information were given to participants in the current pandemic on how to manage their illness during the lockdown, and perhaps compliance monitoring strategies are required to reduce the consequences of the COVID-19 lockdown. There is a need for more studies focused on crises that require a degree of lockdown to establish national compliance values.

## 7. Study Limitations

It is important to mention some limitations. First, considering the extent of the spread of the COVID-19 pandemic, this is a limited scale study. Second, the study findings were drawn from a population in a specific geographic area, and the people were mainly of middle and higher socioeconomic status. Hence, the study findings cannot be generalized to wider populations. Additionally, the sample might be homogenous in term of having the same lifestyles, habits, and characteristics, and this could affect the research outcomes. The study did not include any blood tests for participants, such as the HbA1c test, to assess participants average blood glucose levels over the past three months of the lockdown, and did not correlate such data with participants’ responses. Hence, future studies should focus on the relationship between participants’ behavior and clinical variables. Finally, the approach used to recruit participants may produce sampling bias. Though this limitation was managed by trying to reach the non-responders, bias is nonetheless expected, since social media was used. The commitments of the participants during lockdown may have limited their ability for risk perception and response, for example, homeschooling, caring for elderly relatives, and working from home. 

## 8. Conclusions

The study findings add insight to the theoretical foundations underlying the response of people with diabetes to manage everyday routines and challenges during the COVID-19 pandemic lockdown. The findings may provide an opportunity for nurses to reflect on issues highlighted by the participants to equip them with the required knowledge and skills to support self-care during national emergencies. Devising guidance and support for people with diabetes during lockdown that consider issues such as risk perception, challenges, support, opportunities and responses, are important for easing the process of adapting a coping behavior.

## Figures and Tables

**Table 1:** Interviewees.

No.	Sex	Age	Years of the Disease	Diabetes Type	Interview Length	Subthemes
1	Male	53	5	Type 2	23	Mandatory vaccination
2	Male	31	2	Type 2	16	Maintain physical activities, think about health
3	Male	33	15	Type 1	30	Focus on healthy food, developing complications
4	Male	49	7	Type 2	16	Developing complications, support
5	Male	27	6	Type 1	20	Maintain physical activities, support, developing complications
6	Male	38	21	Type 1	18	Mandatory vaccination
7	Female	46	9	Type 2	15	Focus on healthy food
8	Female	50	13	Type 2	17	Maintain physical activities, think about health
9	Male	61	26	Type 2	13	Maintain balance blood glucose, maintain physical activities
10	Male	57	16	Type 2	19	Infection with COVID-19
11	Male	41	3	Type 2	21	Maintain balance blood glucose
12	Female	22	5	Type 1	25	Infection with COVID-19, think about health, support, developing complications

**Table 2:** Findings.

Themes	Subthemes
Facing our fears	Infection with COVID-19
	Mandatory vaccination
The possibility of damage.	Developing complications
The challenges of everyday routines under lockdown	Maintain balance blood glucose
Identifying strengths	Opportunity to think about health
	Sources of support
Looking for and adapting of alternative solutions for everyday routine	Focus on healthy food
	Maintain physical activities

## Data Availability

All data generated or analyzed during this study are included in this published article.

## Appendix B. Interviews Protocol

Background: Tell me about yourself little bet.

1. What is your age and what type of diabetes do you have?
2. When was the first time you were diagnosed with diabetes?
3. Was your diabetes under control before the lockdown?

Routine diabetes care during lockdown

1. How you see the lockdown?
2. Tell me how do you maintained a healthy lifestyle during the COVID-19 lockdown?
3. Did any of your diet, physical activity and routine check your blood glucose influenced by the lockdown and how? And how did you mange that?
4. Were your medication (insulin/tab) supplies influenced in any way by the COVID-19 lockdown?
5. What was your primary resource(s) of health information during the COVID-19 lockdown?

Diabetes-related worry and concern

1. Were you worried about your health during the COVID-19 pandemic? Does this affect your life?
2. What kind of support did you receive during COVID-19 lockdown? Did the support help you? Why?
3. Are there any other factors that play a role in your ability to accept or refuse changes in your lifestyle during the lockdown?

COVID-19 vaccine perception

1. What do you think about the COVID-19 vaccine? Tell me more about it.
2. Are you willing to get vaccinated? Why?

Time 15 to 30 min
